# An integrative approach to characterize disease-specific pathways and their coordination: a case study in cancer

**DOI:** 10.1186/1471-2164-9-S1-S12

**Published:** 2008-03-20

**Authors:** Min Xu, Ming-Chih J Kao, Juan Nunez-Iglesias, Joseph R Nevins, Mike West, Xianghong Jasmine Zhou

**Affiliations:** 1 Program in Molecular and Computational Biology, University of Southern California, Los Angeles, CA, USA; 2 School of Medicine, University of Michigan, Ann Arbor, MI, USA; 3 Institute for Genome Sciences and Policy, Duke University, Durham, NC, USA; 4 Institute of Statistics and Decision Sciences, Duke University, Durham, NC, USA

## Abstract

**Background:**

The most common application of microarray technology in disease research is to identify genes differentially expressed in disease versus normal tissues. However, it is known that, in complex diseases, phenotypes are determined not only by genes, but also by the underlying structure of genetic networks. Often, it is the interaction of many genes that causes phenotypic variations.

**Results:**

In this work, using cancer as an example, we develop graph-based methods to integrate multiple microarray datasets to discover disease-related co-expression network modules. We propose an unsupervised method that take into account both co-expression dynamics and network topological information to simultaneously infer network modules and phenotype conditions in which they are activated or de-activated. Using our method, we have discovered network modules specific to cancer or subtypes of cancers. Many of these modules are consistent with or supported by their functional annotations or their previously known involvement in cancer. In particular, we identified a module that is predominately activated in breast cancer and is involved in tumor suppression. While individual components of this module have been suggested to be associated with tumor suppression, their coordinated function has never been elucidated. Here by adopting a network perspective, we have identified their interrelationships and, particularly, a hub gene PDGFRL that may play an important role in this tumor suppressor network.

**Conclusion:**

Using a network-based approach, our method provides new insights into the complex cellular mechanisms that characterize cancer and cancer subtypes. By incorporating co-expression dynamics information, our approach can not only extract more functionally homogeneous modules than those based solely on network topology, but also reveal pathway coordination beyond co-expression.

## Introduction

The recent development of microarray technology has significantly facilitated the identification of disease-related genes [1-4]. However, many disease phenotypes are determined not by individual genes, but by the coordinated effect of many genes. Insight into the structure and coordination of disease-related pathways is crucial to understanding the pathophysiology of complex diseases. However, it has proved difficult to infer pathways from microarray data by deriving modules of multiple related genes, rather than individual genes. The major challenges are: (1) Genes involved in a pathway may exhibit complex expression relationships beyond co-expression, which may be overlooked by standard microarray analysis methods such as clustering [5]. (2) Pathways are dynamic and the current static annotation of pathways may not serve as a good template. In fact, pathways are manual dissections of the underlying dynamic gene regulatory network. Under different conditions, different segments of the ensemble network will be activated, leading to condition-specific activation of pathways [6].

**Figure 1:**
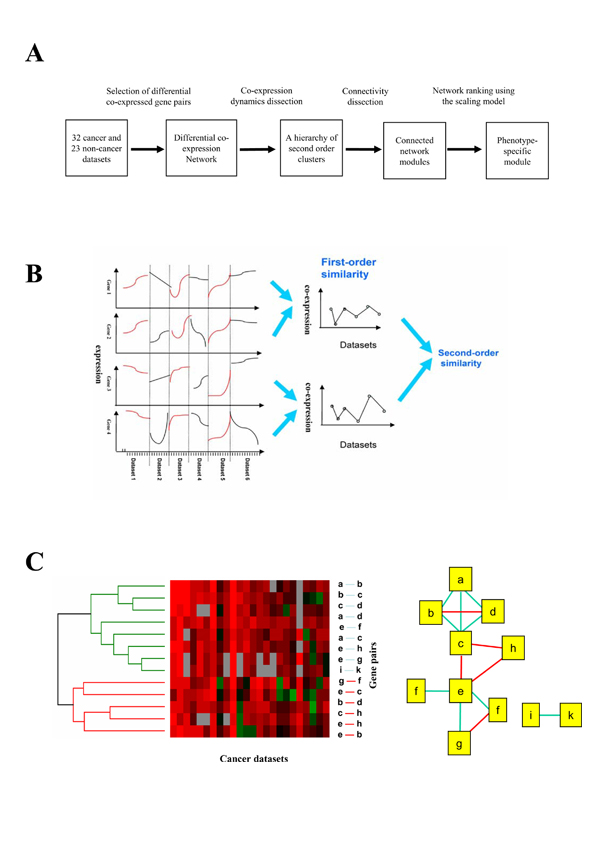
**Overview of analysis procedure**. (A) Flow chart of the analysis pipeline. (B) Schematic illustration of the concept of second-order similarity. It is obvious that the overall expression similarity between the two gene pairs (genes 1 and 2 versus genes 3 and 4) is not significantly high, but their first-order expression correlation profiles exhibit high second-order similarity. (C) Schematic illustration of the dissection of differential co-expression networks into network modules based on the co-expression dynamics and network connectivity. In the heat map, every column corresponds to a dataset and every row corresponds to a gene pair. Red, black, green and grey corresponds to positive, low, negative and missing correlations, respectively. By hierarchical clustering, the gene pairs fall into two major second-order clusters. The 9 gene pairs in the green cluster comprise three connected network components, whereas the 6 gene pairs in the red cluster give rise to three connected components. Furthermore, by considering the second order cluster of a higher level of the hierarchy, which consists of both green and red clusters, the six networks are united to form two connected networks, reflecting the hierarchical modularity of cancer co-expression networks.

In this study, by integrating many microarray datasets we propose a novel method to simultaneously infer pathways and disease/phenotypic conditions under which the pathways are activated. The identified pathways may comprise genes with complex expression relationships beyond co-expression. Due to the existence of a large amount of cancer microarray data, we used cancer as our case study. We collected a series of microarray datasets measuring different types of cancers, and a series of datasets measuring other cellular/physiological conditions. We first construct a differential co-expression network, in which each node represents a gene and each edge indicates a gene pair that is frequently co-expressed in cancer datasets but not in non-cancer datasets. We then dissect the networks into cancer-subtype specific network modules by considering (1) co-expression dynamics and (2) network topology. Figure 1a illustrates the conceptual pipeline of our method.

To measure co-expression dynamics, we use second-order expression similarity, which we proposed previously [5]. Briefly, if we define first-order expression similarity as the expression similarity of two genes from one dataset, then second-order similarity measures whether two gene pairs simultaneously exhibit either high or low expression similarity across multiple datasets. In general, high first-order similarity suggests the existence of a functional link between two genes, and clustering based on the second-order similarity captures multiple functional links always activated and deactivated under similar conditions. Such functional links are likely to comprise a functional module. Interestingly, genes in a second-order cluster may not always have high first-order similarity (see an example in Figure 1b); therefore, second-order analysis allows us to identify functional modules that are inaccessible to co-expression analysis.

Given multiple gene pairs sharing high second-order similarity, we further divide them into network components based on their connectivity on the differential co-expression graph (see an example in Figure 1c). We observe that genes within a connected network component are more likely to participate in the same specific pathway than those between different components (see Supplementary document in Additional file 1), which, in turn, are likely to be involved in different relevant pathways. This may reveal high-order cross-pathway coordination. In fact, hierarchical clustering of differentially co-expressed gene pairs based on their second-order similarity results in a hierarchical modularity in terms of relevance of functional links. We designed a linear scaling model to select modules by considering both module size (number of edges) and within-module second-order similarity. Then, given selected modules, we can further infer datasets (phenotypic conditions) in which a module is activated, i.e. in which genes in the module coordinate.

Applying our methods to 32 cancer-related microarray datasets, and 23 non-cancer related datasets, we derived 162 second-order clusters consisting of 224 network modules, activated either in cancer or in specific cancer subtypes. In particular, we identified a breast cancer specific network module that involved in tumor suppression via platelet-derived growth factor (PDGF)-like signaling, more importantly, a hub gene PDGFRL that may play an important role in this tumor suppressor module.

## Results

### Network properties of the cancer differential co-expression network

We curated 32 human microarray datasets (1,764 expression profiles in total) measuring cancers of 12 tissues, and 23 datasets (1,158 expression profiles) *not* related to cancer (e.g. normal tissues, chronic granulomatous disease, Huntington's disease, inflammatory response). For details of the datasets refer to the Supplementary Table 1 in Additional file 2. We first identify gene pairs which consistently demonstrate higher correlation in cancer versus non-cancer datasets based on a robust correlation estimator, the normalized Percentage Bend correlation (for details see Methods). In following sections, if not specified, the term *correlation* will by default refer to the normalized Percentage Bend correlation. These criteria result in 6,035 gene pairs covering 1,967 genes. The 6,035 gene pairs, each representing a potential conditional functional link, can be represented as a *differential co-expression* network. In this network, each gene is represented as a node and each differential co-expression relationship is represented as an edge.

It has been reported that co-expression networks follow a scale-free node degree distribution [7]. We observed that the differential co-expression network also follows such a topology, where only a small number of nodes act as “highly connected hubs” (see node degree distribution in Supplementary Figure 1 in Additional file 1). This indicates that most gene-gene co-expression relationships differing between cancer and other phenotypes are associated with only a few “hub” genes. Such hub genes exhibit a high degree of coordination with many other genes in neoplastic states, and are therefore likely to play important roles in carcinogenesis and cancer progression. In fact, most hub genes fall into two main functional categories: 1) core processes of neoplastic states such as cell division and chromosome organization; or 2) dynamic interactions between cancer cells and their microenvironment such as angiogenesis, immune response, and cell adhesion (see Supplementary Table 2 in Additional file 2). For those hub genes with unknown functions, we can predict their cancer-related functions based on their neighbor genes. For example, the 16 out of the 33 interacting partners of the ADP-ribosylation factor-like 6 interacting protein (ARL6IP) are involved in cell division (hypergeometric test *p*-value 1.6 × 10^-24^). Thus, ARL6IP is likely to be involved in cell proliferation, consistent with its initial characterization as an interaction partner of the Ras superfamily member ARL6 [8]. As another example, while microfibrillar-associated protein 2 (MFAP2) has long been known to bind to various components of the elastic extracellular matrix [9], it has not been clear whether it serves more than a mechanical function. We found that 6 out of its 24 neighbor genes are involved in cell adhesion (p-value 7.7 × 10^-5^). In fact, a recent study found that MFAP2 binds to a neighbor gene Notch1 and activates it [10].

### Identification of pathway modules specific to cancer or cancer subtypes

The differential co-expression network provides a summary of co-expression links frequently active across all types of cancers. However, it does not provide clues as to which set of links tend to be simultaneously active and inactive under which types of cancer. That is, the edges of a differential co-expression network may not be active in the same subset of datasets. In fact, the largest connected component of the differential co-expression network contains 5944 edges, which comprises 98% of all the edges in the network. Thus, based on connectivity alone we cannot break the network into functionally coherent and cancer-subtype specific modules.

To dissect the networks, we integrate two types of information, the co-expression dynamics and the network connectivity, to extract cancer-subtype specific network modules. First, we employ the second-order clustering approach to utilize the co-expression dynamics information. This includes two steps: (i) for any two genes connected with an edge in the differential co-expression network, we calculate the expression correlation in each of the 32 cancer microarray datasets and store it in a vector, termed the *first-order expression correlation profile* of the genes; (ii) we then perform hierarchical clustering of all the gene pairs based on the Euclidean distance between the first-order expression correlation profiles. Unlike commonly used clustering approaches, the unit of the second-order clustering is a gene pair instead of a gene, and the distance between units is computed based on the first-order expression correlation profiles instead of the original gene expression profiles, hence the term “second-order” clustering [5]. Since each edge represents a frequently occurring co-expression relationship in multiple cancer datasets, it likely represents a functional link. If a cluster of gene pairs follows the same co-expression pattern across multiple cancer datasets, it represents a module of functional links being turned on or off simultaneously across different cancer phenotypes.

Given a second-order cluster of gene pairs, we further identify connected network components among them. We suggest that a set of gene pairs is more likely to be functionally related if they form a connected component (see Supplementary document in Additional file 1 for supporting analysis). Given a second-order hierarchical clustering tree, we traverse the tree bottom up to retrieve connected network components. In general, the size of a connected component (*S*, the number of edges) decreases with the second-order diameter (*D*), defined as the largest pairwise second-order distance. We found that *S* and *D* show a linear scaling relationship in a logarithm scale (see Supplementary Figure 2 in Additional file 1). We are especially interested in outliers – network components small in *D* but large in *S*, which represent tightly clustered network modules relative to their size. We define the modularity score *λ* of a subnetwork using a linear scaling model *λ* = *α* log_2_(*S*) – log_2_(*D*) – *β*, where *α* and *β* are estimated using linear fitting. With our data, we obtain *α* = 0.13 and *β* = 2.2. We select the top 60% of networks (*S* >= 4) ranked by *λ* scores, removing those networks having *D* >= 0.34, and merging heavily overlapping networks. This procedure resulted in 162 second-order clusters comprising 224 connected network modules, with size ranging from 4 to 64 edges. Their composition, network topology, and activation status across various cancer phenotypes can be seen in Supplementary table 3 in Additional file 2. 175 (78%) modules are statistically significantly functionally homogenous based on the GeneOntology Biological Process annotation (hypergeometric test *p*-value <0.01). The most predominant functional categories are cell cycle, cell division, cell proliferation, response to stress, immune response and cell adhesion (see Supplementary Table 4 in Additional file 2), consistent with known pathological mechanisms of cancer.

**Figure 2:**
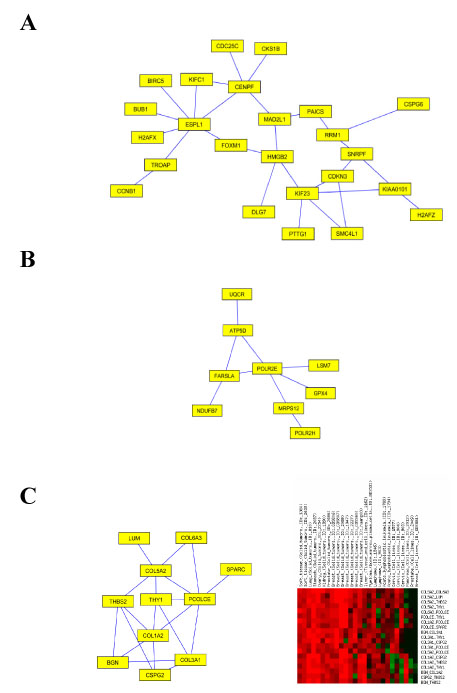
**General cancer and solid tumor network modules.** (A) A network module that tend to activate in most of cancer datasets, consisting 24 genes and 28 edges. Average correlation across all data sets is 0.42. Most of genes in the module are related to cell division and genetic stability. (B) Another network module that is activated in most of cancer datasets, consisting 9 genes and 9 edges. The module is located in the same second order cluster as the one in figure 2a. Its average correlation across all datasets is 0.39. Most of genes in the module are related to nucleobase, nucleoside, nucleotide and nucleic acid metabolism. (C) Left : a network that tends to be activated only in solid tumor datasets. Right, the co-expression heatmap of the edges across datasets. Six datasets are not shown in the heatmap due to lack of valid co-expression estimations. Average correlation in solid tumor datasets and other datasets are 0.61 and 0.17, respectively.

One main feature of our approach is that it can simultaneously discover network modules and the types of cancer in which the modules are activated. Figure 2a shows a module that is activated in most of the cancer datasets. The genes of the module are mostly involved in cell division and genetic stability, representing a cell proliferation signature, a key feature of cancer. Figure 2c shows a network module which tends to be activated in only solid tumors. The genes of the module are mostly involved in cell adhesion and organogenesis, which is specific to solid tumor versus blood cancers or neoplastic cell lines. In the next section, we will detail two network modules which are activated predominately in breast cancer data sets.

### Network modules in the breast cancer cluster

Our analysis resulted in a second-order cluster containing two connected network modules that tend to be more active in all seven breast tumor datasets relative to the rest of the datasets. The average correlation of these modules in breast tumor and other cancer datasets are 0.49 and 0.23 respectively (the *t*-test of co-expressions between breast tumor and the rest of cancer datasets gives a *p*-value of 1.56 × 10^-95^).

#### A tumor suppressor network related to PDGF superfamily signaling

**Figure 3:**
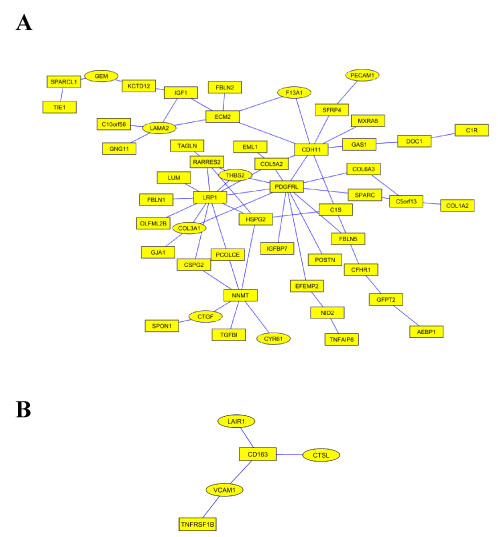
**Coordinated breast tumor network modules** (A) A breast tumor network module that involved in PDGF signalling. Genes in round shape have promoter regions that are predicted to be bound by transcription factor ZNF148. (B) A breast tumor network module related to inflammatory response, located in the same second-order cluster as the one in Figure 3a. Genes in round shape have promoter regions that are predicted to be bound by transcription factor POU2F1.

The module in Figure 3a contains 52 genes. Most of them are extracellular or membrane proteins, and 23 genes have previously been found to be involved in breast cancer. A number of such examples are listed in Supplementary document in Additional file 1. Among the 52 genes, 16 are involved in cell adhesion (p-value 7.4 × 10^-11^), and 14 are involved cell-cell signalling (p-value 7.9 × 10^-6^), suggesting a role in tumor invasiveness of the module [11].

Most interestingly, we found one main function of this network module appears to be tumor suppression via the inhibition of PDGF superfamily signaling. A hub gene with high degree of this module is the gene PDGF receptor-like (PDGFRL) (degree 11). While its precise biological function is not known, PDGFRL encodes a 375aa product with significant sequence similarity to the extracellular domain of PDGFR. Indeed, mutations in PDGFRL have been found in individual cancer samples [12-15]. PDGFRL is located in chromosome 8p22-8p21.3, where multiple studies have suggested the existence of a putative breast cancer tumor suppressor gene [16], [17], [18]. Recently, an in-depth study of the region using microcell-mediated chromosome transfer found that indeed PDGFRL expression is decreased in the majority of breast cancer cells [19]. Many genes in this network module have been found to be involved in PDGF superfamily signaling. For example, Cysteine-rich protein (SPARC) binds to PDGF-AB and PDGF-BB dimers, inhibiting the binding of these growth factors to their cell surface receptors [20] and inhibiting PDGF-induced vascular smooth muscle proliferation [21]. Also, connective tissue growth factor (CTGF) has structural similarities with PDGF [22]. Recently, two new PDGF ligands were discovered that have a N-terminal complement subcomponent C1R/C1S, UEGf, BMP1 (CUB) domain [23]. Interestingly both C1R and C1S are members of this network module. Also, LRP1 is a physiological modulator of the PDGF signaling pathway [24]. In total, we found direct Pubmed literature support for 19 out of the 52 member genes to be involved in (or related to) the PDGF-superfamily signalling. Furthermore, it is known that zinc finger protein (ZNF148) binds to the PDGFR [25] gene promoter. We screened the promoter regions of the 52 member genes, and found that the binding sites of ZNF148 are significantly enriched (hypergeometric *p*-value 0.016). In addition, it has been reported that PDGFR positively regulates collagen production (for example [26], [27]). Our module showed the breast tumor specific co-expression between PDGFRL and collagens COL3A1, COL5A2 and COL6A3. Many of the above evidences support the hypothesis that PDGFRL has an agonistic function to PDGFR signaling.

Although abundant evidence suggests the individual involvement of many of these genes in tumor suppression, their coordinated function has never been elucidated. Here by adopting a network perspective, we have identified their interrelationships and a gene (PDGFRL) that may play a central role in this tumor suppressor network.

#### A network module related to inflammatory response

Another identified breast cancer-specific network module (Figure 3b) may be involved in the coordination of the inflammatory response to cancer pathology. This network consists of 5 genes: tumor necrosis factor receptor superfamily member 1B (TNFRSF1B); vascular cell adhesion molecule 1 (VCAM1); leukocyte-associated immunoglobulin-like receptor 1 (LAIR1); and Cathepsin L1 (CSTL). They are arranged around the macrophage-associated antigen (CD163). Several lines of evidence have implicated these genes individually in breast cancer, for example: increased plasma levels of VCAM1 is associated with advanced breast cancer [28]; genetic variation in TNFRSF1B may predict the late onset of breast carcinoma, and relapse and death for patients with breast carcinoma [29]; finally, the breast cancer cell line exhibiting the highest in vitro invasiveness also expressed the highest amount of CTSL1 splice variant L-A3 [30].

Most of the genes of this module are related to tumor necrosis factor (TNF), an inflammatory cytokine. It has been reported that activation of rat CD163 on peritoneal macrophages induces the production of pro-inflammatory mediators including TNF [31]; TNF directly interacts with TNFRSF1B [32] and is a mediator of TNF function in the mouse ovary [33]. Finally, transcription of VCAM1 in endothelial cells can be induced by TNF [34].

A transcription factor, octamer-binding transcription factor-1 (POU2F1, also known as Oct-1) has been predicted to bind promoter region of VCAM1, LAIR1 and CTSL (hypergeometric *p*-value 0.012). The binding of POU2F1 to VCAM1 promoter is indeed supported by the literature [35-37]. Also, POU2F1 has been found to bind SP3 [38], which is reported to activate the transcription of CSTL [39].

The tight and coordinated expression of the genes in this network module reveals an induced inflammatory response that may be important in breast tumor progression [40].

### Identification of pathway coordination beyond co-expression

A major advantage of second-order clustering is that it can identify functionally related genes beyond co-expression, as illustrated in Figure 1b. We elaborate on this point in this section. To allow readers to easily assess the magnitude of the correlation, only in this section we use the Pearson correlation to measure the co-expression level.

Based on the definition of second-order clustering, connected network components within the same second-order cluster show coordinated activities, which implies their functional relevance. In the example of the two modules in the general cancer cluster in Figure 2a and 2b, each module may play different roles in the regulation of specific biological processes -- cell division and nucleic acid metabolism, respectively; the latter is clearly required for cell division. Given the fact that these processes belong to the same second-order cluster, they may represent facets of the same underlying neoplastic process. However, member genes of the two modules exhibit distinct expression patterns: The average Pearson correlation between genes of the two modules is only 0.13.

As another example, the two breast cancer modules described in last section are also related. Indeed, the collaboration of PDGF signalling and TNF have long been known to be required for tissue repairing [41], and their abnormal expression play important but partially defined roles in breast tumor development and progression [42]. On the other hand, member genes of the two modules exhibit relatively distinct expression patterns. For example, two hub genes of modules, PDGFRL and CD163, also show very weak expression similarities across all seven breast cancer datasets: the average Pearson correlation between these two genes is only 0.24.

Besides the above examples, we found coordinated modules within the majority of identified second-order clusters. Overall, from the total 162 second-order clusters, 25% give rise to more than one connected network module. To estimate the amount of cross module co-expression within second-order clusters, for each second order cluster, we first determined the active cancer datasets, in which the average Pearson correlation of gene pairs in the cluster is greater than 0.5. For 72% of those module pairs within the same second-order cluster, the average gene pairwise Pearson correlation between modules in the active datasets is less than 0.5 (normalized Percentage Bend correlation approximately < 0.35), and for 30% module pairs the cross-module average gene pairwise Pearson correlation is less than 0.3 (normalized Percentage Bend correlation approximately < 0.19).

Furthermore, even genes in the same network module are not necessarily highly co-expressed when the module is active, despite their high degree of functional homogeneity, as discussed previously. We found that in 32 of 224 (14 %) modules, the average pairwise Pearson correlation of any two genes in the module is < 0.5 in the corresponding active datasets. Such modules could therefore easily be overlooked by traditional clustering methods.

## Discussion

The rapid accumulation of microarray data provides unprecedented opportunities to study the molecular mechanisms underlying disease pathogenesis and progression. Although many studies utilized multiple microarray datasets to derive consistent lists of genes specific for (subtypes of) cancer [43-45], little attention has been paid to derive genetic networks characterizing different types of cancer. Segal et al. [46] used predefined biologically meaningful gene sets including known biological pathways, and have successfully identified activated or repressed biological modules in a wide variety of neoplastic conditions. The approach, however, relies on the knowledge of pre-defined biological modules and has limited use in the discovery of novel association between genes. A recent study by Choi et al. [47] compared the two co-expression networks summarized from 10 tumor and normal datasets, respectively, and have identified functional differences between normal growth and cancer in terms of gene coexpression changes in broad areas of physiology. However, due to the multifaceted nature of cancer, interactions in such a derived summary network may not be simultaneously active in individual datasets, i.e. specific cancer conditions.

In this study, we propose an unsupervised method that integrates both co-expression dynamics and network topology information to characterize cancer (subtype) specific network modules. The identified modules, such as modules activated across all cancer subtypes or only in solid tumors, are novel, but consistent with known molecular mechanisms. Importantly, we have discovered a potential tumor suppressor network particularly active in breast tumors, and provide compelling evidence that the hub gene PDGFRL is a true tumor suppressor gene. Compared to commonly used differential or co-expression analysis, our approach has the following advantages: (1) our unsupervised approach simultaneously discovers network modules and the conditions (e.g. cancer subtypes) in which they are activated, thus providing new insights into the complex cellular mechanisms that characterize cancer and cancer subtypes. (2) Compared to existing approaches [47-51] which can only identify densely connected network modules based solely on network topology information, our approach incorporating co-expression dynamics information (second-order similarity) can extract more diverse types of modules regardless of network density. It is known that many biological pathways do not necessarily form densely connected modules. (3) Our approach can reveal coordination of pathways beyond co-expression. (4) Our method can be applied to any types of molecular networks beyond co-expression network, when data of multiple networks under different conditions are available.

In the current framework, the selection of biologically meaningful modules still need certain amount of manual intervention from biology expert. We are looking for more systematic ways for module selection, especially by putting the framework into the context of network statistics to improve the robustness. For example, although the scaling model we constructed is based on direct observation of the distribution of network properties S and D, their log-linear relationship suggests that it should be straightforward to make use of the exponential random graph models that have been used in recent years to study statistical aspects of networks [52]. In essence, such models linearly combine network properties and assign the probability of observed networks as the exponential of such linear combinations. Integrating this work with the exponential family probabilistic models may provide both better estimation of the coefficients in the linear scaling model and more accurate selection of network modules via hypothesis testing. We intend to explore this direction in future work.

The choice of datasets depends on the research question. Ideally there should be a balanced and sufficient sampling of different phenotypes (in particular different tissues for this cancer study). Particularly, a paired Wilcoxon test between cancer and normal samples of the same tissue would significantly eliminate the amount of tissue-specific co-expressions. However, due to the limited amount and the heterogeneity of existing data it is currently impractical to achieve this goal. Using a weighted sampling scheme could potentially bypass the imbalance effects. We aim to investigate this strategy, and use statistical models of the correlation values to determine the weighting factors.

## Methods

### Datasets

We curated 32 cancer and 23 non-cancer human gene expression datasets mainly from the Stanford Microarray Database (SMD) and Gene Expression Omnibus (GEO) databases, each containing more than 15 microarrays, on either Affymetrix or cDNA platforms. In each dataset, if there are multiple probes that correspond to the same gene, we choose the one that contains the least amount of missing values. For datasets containing absolute expression measurements, we convert all values <= 10 to 10, then perform a base 2 log transform.

### Estimation of Pairwise gene co-expression

We used Percentage Bend Correlation [53] (with *β*=0.05) to obtain a robust correlation estimate. Percentage Bend Correlation first detects outliers in expression values of each gene then reduces the effects of those outliers in the correlation calculation. Only gene pairs with a large number of valid samples *m*>= 15 are used to calculate correlation. To make the correlation estimates comparable across different datasets of variable sample sizes and among different gene pairs of different amount of missing expression measures within the same dataset, we performed Fisher's *z*-transform [54] to reduce sample size effect. Given a correlation estimate *r* and sample size *n*, the Fisher's *Z* scores (divided by its theoretical standard deviation) is calculated as $z=\frac{\sqrt{n-3}}{2}\log\left(\frac{1+r}{1-r}\right)$, which theoretically has an asymptotically standard normal distribution. Note that sample size *n* may be different from gene pair to gene pair due to missing values, and from dataset to dataset. In reality, we observed that the distributions of the z-score are still different from dataset to dataset: we therefore normalized z-scores to enforce the standard normal distribution. After that, standardized correlations *r'* are obtained by inverting the z-score with a fixed *n* of 30.

### Select differentially co-expressed gene pairs

We define a gene pair to be differentially co-expressed between cancer and non-cancer if it satisfies the following two criteria: (1) their expression correlation in cancer datasets is sufficiently strong (can be either positively or negatively high). This is done by setting threshold for average summed square of correlations in cancer datasets. i.e, $\sqrt{\frac{1}{c}\sum_{k}{\left(r_{i}^{\left(k\right)}\right)}^{2}}\geq 0.35$ for the gene pair *i*, where there are *c* valid correlation estimations (c=32 if there are no missing values) and *k* is dataset index corresponding to all valid correlation estimations; and (2) the Wilcoxon ranksum test of correlations between cancer and non-cancer datasets gives a *p*-value <= 0.01.

### Identify conditionally activated network module candidates

We hierarchically clustered the differentially co-expressed gene pairs based on their expression correlation profiles using the CLUSTER program [55] with complete linkage and Euclidean distance. The Euclidean distance is averaged to provide a simple estimation given the existence of missing correlations (due to the missing value problem). $d_{i,j}=\sqrt{\frac{1}{c}\sum_{k}{\left(r_{i}^{\left(k\right)}-r_{j}^{\left(k\right)}\right)}^{2}}$ where *r_i_^(k)^* and *r_j_^(k)^* are the correlations of gene pairs *i* and *j* in the dataset *k*, respectively, and *c* is number of valid correlations. In the hierarchical tree, each leaf node represents a gene pair, and each inner node corresponds to a second-order cluster of gene pairs (edges) which may comprise zero, one, or more connected network components. In cases where the size of the differential network is too big to be processed using hierarchical clustering (HC), the gene pairs were first separated using k-means clustering then processed the smaller clusters separately by hierarchical clustering. As for our experience, the biologically meaningful modules normally contain less than a few hundred edges, thus k-means clustering will keep most of modules intact.

### Gene ontology function and transcription factor enrichment of modules

The functional enrichment analysis is done by the hypergeometric test on genes. We selected 419 Gene Ontology (GO) functions (i.e. biological process terms) which are 4 levels below the root in the GO hierarchy. Each gene may be directly or indirectly associated with some of these functions. A set of genes will be considered to have a enriched function when (1) the functional homogeneity modeled by the hypergeometric distribution [56] is significant at a significance level 0.01 and (2) there are at least 2 genes in the set are associated with the function.

### Identification of transcription factor binding

10kb upstream sequences for each gene were obtained from NCBI Gene database. After applying RepeatMasker [57], we used the MATCH program of TRANSFAC [58] (version 9.2) to scan the sequences for the presence of transcription factor binding sites based on position weight matrices. We used vertebrate-specific matrices, and chose cut-offs to minimize the sum of false positives and false negatives. We kept only the top 3,000 hits per matrix, sorted by the matrix similarity score. Altogether we obtained 349,178 predicted transcription factor target relationships of 180 transcription factors. A hypergeometric test was performed for each network module to search for over-represented transcription factor binding sites.

## Competing interests

The authors declare that they have no competing interests.

## Authors' contributions

XJZ conceived the research problem. MX and XJZ designed the methodology. JNI and MW provided suggestions for the design. MX developed programs, collected and processed data. JNI curated data. XJZ, MX and JNI provided general interpretation of results. MJK, XJZ and JNI analyzed specific examples. MX, XJZ, MJK, and JNI drafted the manuscript. JRN commented on the manuscript. MW and JRN suggested more applications of the method. All authors read and approved the final manuscript.

## Supplementary Material

Additional file 1 This document includes 1) description of selection of hub genes, 2) analysis of functional network homogeneity, 3) description of supplementary tables and 4) supplementary figures. Click here for file

Additional file 2 Click here for file
