# Supplementary material for: An integrative approach to characterize disease-specific pathways and their coordination: a case study in cancer
Source: BMC Genomics. 2008 Mar 20;9(Suppl 1):S12. doi: 10.1186/1471-2164-9-S1-S12 (PMC2386054; doi:10.1186/1471-2164-9-S1-S12)
Supplement: Additional file 1 — This document includes 1) description of selection of hub genes, 2) analysis of functional network homogeneity, 3) description of supplementary tables and 4) supplementary figures. [file 1471-2164-9-S1-S12-S1.PDF]

Supplementary Figures

Figure 1: node degree distribution on the differential co-expression networks.

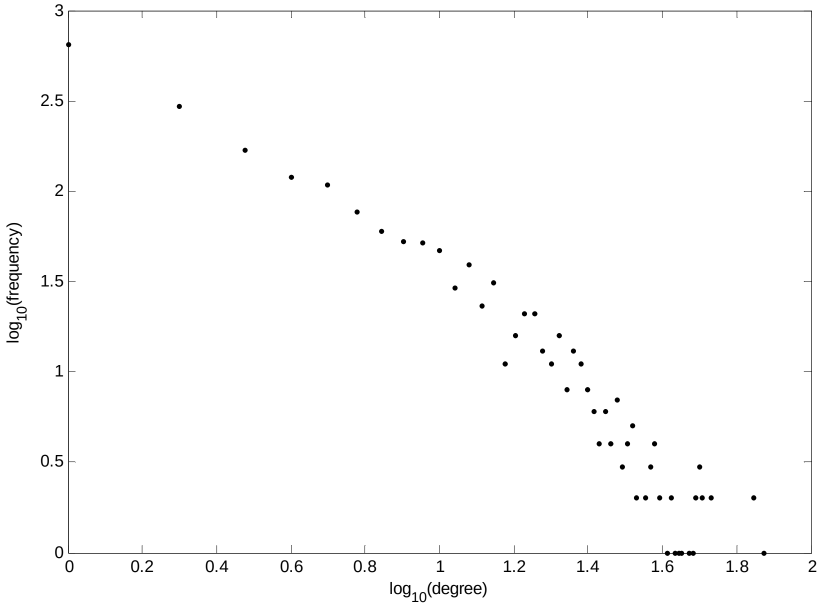

Figure 2: The linear relationship between network size S and cluster diameter D. Each dot in the figure corresponds to a connected network of size >= 4. The line is fitted using networks with size >= 10.

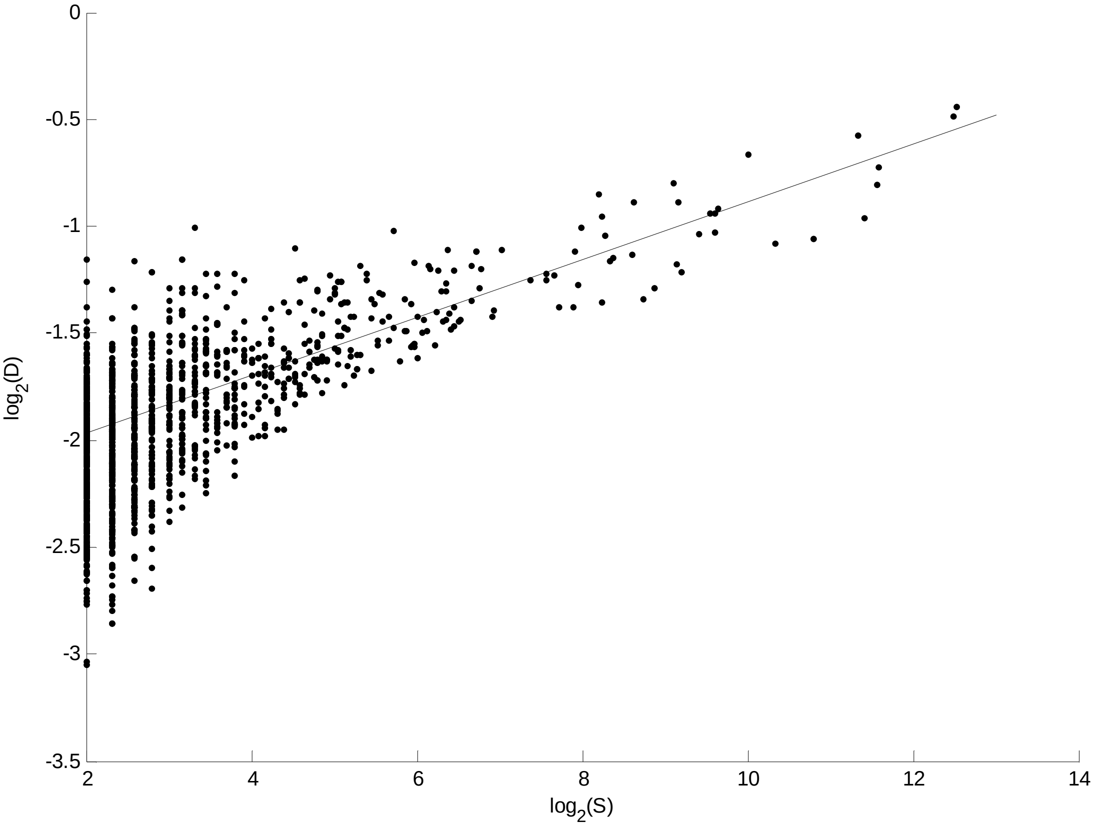

Supplementary Tables :

Table 1, description of datasets, including both cancer and non-cancer datasets. Originally 51 microarray datasets are downloaded mainly from SMD (with four digit dataset ids) and GEO (dataset id starts with ‘GDS’). We divide a dataset if it contain both cancer and non-cancer samples. The sample numbers that highlighted in yellow correspond to datasets that are kept to form 32 and 23 cancer and non-cancer datasets for our study.

Table 2, 112 selected hub genes. Their degrees, Gene Ontology annotations, the functional enrichment of neighbor genes and whether the hub gene is known to relate cancer.

Table 3, selected network modules and their edge average co-expression in cancer datasets.

Table 4. Enriched functions in modules. The enrichment is tested by hypergeometric test p-value < 0.01.

In the differential networks, we selected the top 112 hub genes that have degrees >= 22, such that they together account for 30% of the total degrees on the differential co-expression networks (supplementary table 2). Many of those genes fall into two main functional categories: 1) core processes of neoplastic states such as cell division and chromosome organization (36 genes); or 2) dynamic interactions between cancer cells and their microenvironment such as angiogenesis, immune response, and cell adhesion (24 genes). Genes falling into category (1) include TUBB (participates in microtubule-based movement; node degree 70), CKS1B (cell cycle; node degree 43), KPNA2 (regulation of DNA recombination; node degree 44), RRM1 (DNA replication; node degree 41), MAD2L1 (cell cycle; node degree 49), and SOX2 (establishment/maintenance of chromatin architecture; node degree 30). Genes in category (2) include MAP2K7 (response to proinflammatory cytokines; node degree 50), TYROBP (cellular defense response; node degree 37), CD4 (immune responses; node degree 45). They are all reported to play a role in cancer pathogenesis and progression. In addition, hub genes in the category (1) behave as hub genes across most of datasets, while those in the category (2) tend to be in hub genes only in solid tumor datasets.

Examples of genes in the breast tumor suppressor modules that known to related to breast cancer : connective tissue growth factor (CTGF) is over-expressed in breast cancer cell lines with increased metastatic activity [1] ; Fibulin 1 (FBLN1) is implicated in immune response against breast cancer [2], and one of its splice variants is over-expressed in breast [3]; the cysteine-rich secreted protein (SPARC) plays a crucial role in tumor development in breast cancer [4]; GAS1 is also found to be induced in apoptotic mammary gland cells [5]; Cysteine-rich angiogenic inducer 61 (CYR61) is involved in the proliferation, cell survival, and Taxol resistance of breast cancer [6]; finally, in the case of a hub gene LRP1 (low density lipoprotein receptor-related protein 1), the T allele of the C766T polymorphism is associated with an increased risk of breast cancer development [7].

The analysis of the functional homogeneity within connected networks vs between networks of the same second-order cluster. The disjoint network modules of same second-order cluster generally fall into different functional categories. From the 162 second-order clusters, we measure the functional similarity of genes within each connect network, and between networks of the same cluster using number of shared Gene Ontology functions. A t-test of the distinction in functional similarities gives p-value 1.6×10^{-8} on our selected network modules shows that the genes tend to be significantly more functionally coherent within a network than between networks. Thus each network is more likely to represent a pathway. But in addition, these networks are in the same second-order cluster, this indicates that these networks are activated together in certain phenotypes.

For module level and cluster level gene function similarity comparison, instead of using the GO functions of a fixed level as in the previous paragraph, for each gene we collect all GO functions (including GO terms directly associated with the gene and all ancestor terms on the GO hierarchy). We measure functional similarity of a set of genes by the number of GO functions shared by the genes. This measure is adapted from [8]. A one tailed paired t-test is performed on functional similarity of all genes within a module versus similarity of all genes in both this module and another module of the same second order cluster.

References :

1. Kang Y, Siegel PM, Shu W, Drobnjak M, Kakonen SM, Cordon-Cardo C, Guise TA, Massague J: **A multigenic program mediating breast cancer metastasis to bone**. *Cancer Cell* 2003, **3**(6):537-549.
2. Pupa SM, Argraves WS, Forti S, Casalini P, Berno V, Agresti R, Aiello P, Invernizzi A, Baldassari P, Twal WO *et al*: **Immunological and pathobiological roles of fibulin-1 in breast cancer**. *Oncogene* 2004, **23**(12):2153-2160.
3. Bardin A, Moll F, Margueron R, Delfour C, Chu ML, Maudelonde T, Cavailles V, Pujol P: **Transcriptional and posttranscriptional regulation of fibulin-1 by estrogens leads to differential induction of messenger ribonucleic acid variants in ovarian and breast cancer cells**. *Endocrinology* 2005, **146**(2):760-768.
4. Watkins G, Douglas-Jones A, Bryce R, Mansel RE, Jiang WG: **Increased levels of SPARC (osteonectin) in human breast cancer tissues and its association with clinical outcomes**. *Prostaglandins Leukot Essent Fatty Acids* 2005, **72**(4):267-272.
5. Seol MB, Bong JJ, Baik M: **Expression profiles of apoptosis genes in mammary epithelial cells**. *Mol Cells* 2005, **20**(1):97-104.
6. Menendez JA, Vellon L, Mehmi I, Teng PK, Griggs DW, Lupu R: **A novel CYR61-triggered 'CYR61-alphavbeta3 integrin loop' regulates breast cancer cell survival and chemosensitivity through activation of ERK1/ERK2 MAPK signaling pathway**. *Oncogene* 2005, **24**(5):761-779.
7. Benes P, Jurajda M, Zaloudik J, Izakovicova-Holla L, Vacha J: **C766T low-density lipoprotein receptor-related protein 1 (LRP1) gene polymorphism and susceptibility to breast cancer**. *Breast Cancer Res* 2003, **5**(3):R77-81.
8. Lee HK, Hsu AK, Sajdak J, Qin J, Pavlidis P: **Coexpression analysis of human genes across many microarray data sets**. *Genome Res* 2004, **14**(6):1085-1094.
